# Compression aids improve cardiovascular control during verticalization in patients after cervical spinal cord injury

**DOI:** 10.3389/fnins.2026.1922241

**Published:** 2026-09-10

**Authors:** Jana Svacinova, Katarina Ondrusova, Zuzana Novakova, Michal Javorka, Marie Novakova

**Affiliations:** 1 Department of Physiology, Faculty of Medicine, Masaryk University, Brno, Czechia; 2 Department of Physiology, Comenius University in Bratislava, Jessenius Faculty of Medicine, Martin, Slovakia

**Keywords:** Baroreflex, causal analysis, cervical spinal cord injury, compression aids, orthostatic hypotension

## Abstract

**Purpose:**

Patients after complete cervical spinal cord injury suffer from autonomic dysregulation. Impaired sympathetic control over cardiovascular system leads to the blood redistribution and in consequence to the hypotension during orthostatic challenge. Compression aids are used as a non-pharmacological therapy supporting venous return and thus preventing hypotension. The aim of the study was to evaluate the effect of CA on the baroreflex control over the cardiovascular system during verticalization.

**Methods:**

Blood pressure was continuously recorded by volume-clamp photoplethysmography in 10 subjects with cervical spinal cord injury in three situations: sitting, verticalization with and verticalization without compression aids. From the blood pressure record, sequences of beat-to-beat systolic, diastolic and pulse pressures, and inter-beat intervals were detected. Causal coherence from systolic blood pressure to inter-beat-intervals and baroreflex sensitivity were calculated by autoregressive bivariate model.

**Results:**

All assessed hemodynamic variables significantly decreased during verticalization and this decrease was attenuated by compression aids application. Coherence did not change during verticalization without compression aids, but increased with compression aids.

**Conclusion:**

The results indicate that application of compression aids supported venous return and consequently stroke volume (reflected by pulse pressure increase). Parasympathetic control related indices (baroreflex sensitivity and respiratory sinus arrhythmia magnitude) decreased during verticalization and this decrease was attenuated by compression aids indicating less prominent cardiac parasympathetic control decrease during verticalization preserving wider functional reserve in reflex baroreflex control to blood pressure perturbations.

## 1 Introduction

Patients after cervical spinal cord injury (SCI) often suffer from both the loss of motor and sensory functions and autonomic dysregulation. Combination of these factors significantly affects cardiovascular system control and results in resting and orthostatic hypotension in the chronic phase of SCI (Claydon et al., 2006).

Orthostatic hypotension (OH) is defined as a decrease by 20 mmHg in systolic blood pressure (SBP), or by 10 mmHg in diastolic blood pressure (DBP), upon the assumption of an upright posture from a supine position. It is present in approximately 74% of patients after cervical SCI and negatively affects their quality of life (Illman et al., 2000). Verticalization as an important part of physiotherapy and mobilization shows positive impact on physical and psycho-social conditions of these patients. However, orthostasis-related symptoms associated with OH can negatively interfere with this important maneuver in the rehabilitation process of SCI patients (Claydon et al., 2006).

Orthostasis is accompanied by an impaired venous return due to blood pooling in the lower parts of the body. As a result, arterial blood pressure (BP) decreases evoking several high-pressure baroreflex mediated compensatory mechanisms to prevent BP drop and thus to preserve blood flow through the vital organs (Krassioukov and Claydon, 2006). Baroreflex mediated response to BP decrease comprises several effects—increased heart rate, increased cardiac contractility, venoconstriction, and vasoconstriction in arterioles (Joyner and Limberg, 2014; Krohova et al., 2020).

In SCI patients, orthostasis-evoked response is compromised at various levels. Although parasympathetic nerves controlling heart rate are anatomically intact and thus well-preserving heart rate response to orthostasis, cervical SCI interrupts sympathetic neural pathways, which results in a loss of supraspinal control over the cardiac contractility and arterioles in the splanchnic and lower extremities circulation (Schmid et al., 1998; Weaver et al., 2012). Similarly, sympathetic control of venous blood capacity is impaired which leads to an excessive blood pooling in SCI patients. Together with the lack of muscle pump activity in the legs and loss of motor control over abdominal muscles (both supporting venous return), the blood pooling in the dependent parts of body becomes more expressed in cervical SCI patients increasing the risk of OH (Popa et al., 2010; West et al., 2012).

Compression aids (CA)—over-knee stockings, abdominal binder or anti-G suits—are used in situations when decreased venous capacity and improved venous return are beneficial for a patient. Compression over-knee stockings are used for non-pharmacological prevention of venous insufficiency and deep vein thrombosis in lower limbs (Agu et al., 1999; Ibegbuna et al., 1997; Prandoni et al., 2004). Compression stockings reduce venous wall distension and improve valvular function, which leads to an increased blood flow from veins towards the heart (Gillis et al., 2008). Considering a decreased venous return in SCI patients associated with an impairment of venous sympathetic control, compression stockings were found to improve exercise performance and to reduce post-exercise hypotension, venous capacitance during orthostasis and the risk of OH (Popa et al., 2010; Hopman et al., 1992; Houtman et al., 1999; Rimaud et al., 2007; Rimaud et al., 2008; Rimaud et al., 2012). It was also demonstrated that abdominal binder supports respiration, which may improve venous return in SCI patients (West et al., 2012; Gillis et al., 2008).

We hypothesize that CA should increase venous return and at least partially prevent the negative effect of verticalization on the hemodynamics and cardiovascular autonomic nervous control of the SCI patients. Therefore, the aim of this study was to assess the effect of CA on the basic hemodynamic measures before and during verticalization. Furthermore, we compared the effect of CA application on cardiac parasympathetic control shifts associated with verticalization in cervical SCI patients.

## 2 Materials and methods

### 2.1 Subjects

Patients with transversal spinal cord lesion in segments C5-C7 of trauma etiology were included in this study. Patients were clinically classified as having complete or incomplete neurological loss, based on the presence/absence of perianal sensory function in accordance with the “International Standards for Neurological Classification of Spinal Cord Injury (ISNCSCI)” (Kirshblum et al., 2011). All patients were classified as AIS A (Krishna et al., 2014). In total, ten patients (9 men, 1 woman) aged 18–35 years were included. Time interval from SCI varied from 2 to 12 years, i.e., all examined subjects were in the chronic stage of SCI and they were stabilized, without any acute health problems and without any medication potentially affecting cardiovascular system or autonomic nervous system activity. Prior to the examination, all subjects were asked to restrict from their diet coffee, black tea and other drinks containing caffeine. Before the measurement, patients catheterized themselves to empty the urinary bladder. Basic study group characteristics are summarized in Table 1.

**Table 1:** Basic characteristics of the studied group.

Patients (*n* = 10)	Median (min—max)
Age (years)	27.5 (19–36)
Body mass (kg)	73 (55–88)
Height (cm)	184 (176–190)
BMI (kg m^−2^)	21.4 (17.8–26.8)

The study was approved by the Ethic committee of Faculty of Medicine, Masaryk University Brno, Czech Republic. All participants signed informed consent based on *“World Medical Association Declaration of Helsinki Ethical Principles for Medical Research Involving Human Subjects*” and they were informed about the purpose of this study and about employed examination approaches.

### 2.2 Study protocol

The study protocol consisted of three phases:

Phase 1: 10 min at rest in sitting position on a wheelchair without compression aids,
Phase 2: 8–10 min in the orthostatic phase after verticalization without compression aids,
Phase 3: 8–10 min in the orthostatic phase with compression aids.

Between phases 2 and 3, at least 10 min resting (recovery phase) on a wheelchair was inserted. Phases 2 and 3 were performed randomly and repeatedly, in order to obtain sufficiently long recordings of a good quality. The recordings with the minimal presence of artifacts were selected for further analysis.

During each phase, arterial BP was measured continuously using the non-invasive volume-clamp plethysmography method (Finometer, FMS, Netherlands). Measurement was performed in a quiet room with a temperature approx. 22 °C. Compression aids consisted of abdominal binder and elastic compression stockings (Loana Lonaris Cotton, Czech Republic, 19–21 mmHg). Upright position during orthostatic phases was ensured by verticalizer Balance Thera-trainer (Medica Medizintechnik GmbH, Germany). When upright, the subjects were asked to report the eventual presence of pre-syncopal symptoms (light-headedness, dizziness, blurred vision, etc.). In case of their presence, the patient was immediately returned back to the sitting position.

### 2.3 Data analysis

Values of beat-to-beat systolic and diastolic BP and inter-beat interval were detected from the continuous finger arterial BP signal. Inter-beat interval (IBI) was defined as a time interval between two neighboring local BP minima corresponding to diastolic BP. Pulse pressure (PP) was calculated as a difference between SBP and DBP value in the given cardiac cycle. Using this approach, beat-to-beat sequences of SBP values (sbp = [sbp_1_, sbp_2_, sbp_3_, …, sbp_n_]), DBP values (dbp = [dbp_1_, dbp_2_, dbp_3_, …, dbp_n_]), IBI values (ibi = [ibi_1_, ibi_2_, ibi_3_, …, ibi_n_]), and PP values (pp = [pp_1_, pp_2_, pp_3_, …, pp_n_]) were obtained. The sbp and ibi time series were aligned as follows: the sbp_i_ value occurred inside the i-th IBI (ibi_i_). For further analysis, 300 beats-long simultaneous sequences of SBP, DBP, IBI, and PP were selected based on the visual inspection of the most stationary segment of recording with the minimum of artifacts. Mean values of SBP, DBP, IBI, and PP were calculated from the 300-beats long sequences of sbp, dbp, ibi, and pp, respectively.

Heart rate response to BP is mediated by baroreflex and sensitivity of this—cardiac chronotropic—baroreflex arm represents an index of reflex parasympathetic activity mediated response. Considering the bidirectional closed-loop interaction between SBP and IBI, baroreflex function was analyzed from the directed interaction sbp → ibi (baroreflex mediated interaction between sbp (source) and ibi (target) oscillations) separately from the opposite non-baroreflex interaction (from ibi to sbp). Applying linear bivariate autoregressive model, two frequency-dependent variables were calculated: causal coherence and gain. Coherence Coh_sbp → ibi_ represents the strength of unidirectional coupling from sbp to ibi. Gain of transfer function from sbp to ibi represents baroreflex sensitivity index (BRS_sbp → ibi_) and quantifies the magnitude of IBI response to a unit change in SBP. Both variables were computed as a mean value in the low frequency band (0.04–0.15 Hz) to minimize the influence of respiratory sinus arrhythmia related mechanisms on sbp → ibi interactions (Malliani, 1999; Malpas, 2002). In addition, mean power of IBI oscillations in the high frequency band 0.15–0.5 Hz (HF IBI) was calculated as a measure of phasic parasympathetic activity (Malliani, 1999).

### 2.4 Statistics

Due to the non-Gaussian distribution of variables, non-parametric analysis tests were applied. The effect of phases was analyzed using Friedman test followed by post-hoc Wilcoxon test. *p*-values below 0.05 were considered statistically significant. Statistical analysis was performed using STATISTICA software (StatSoft, version 10, Tulsa, OK, USA).

## 3 Results

Five SCI patients reported relatively strong OH-related symptoms during verticalization, subjectively weakened by CA use. These symptoms were highly reproducible during repeated verticalizations. In the second half of patients, no OH-related symptoms were reported. To prevent a marked decrease in a power of the following statistical analysis, we have not divided the study sample into symptomatic and non-symptomatic patients and we consider our group of subjects as one study sample. During a detailed analysis of the results, no statistically significant correlations were found between the level of the spinal cord lesion and any of the monitored parameters (HRV, coherence, blood pressure, or BRS). One subject displayed markedly higher baroreflex sensitivity in the sitting position than the other participants. Despite this difference, the response to verticalization was comparable to that observed in the rest of the study group.

**Figure 1:**
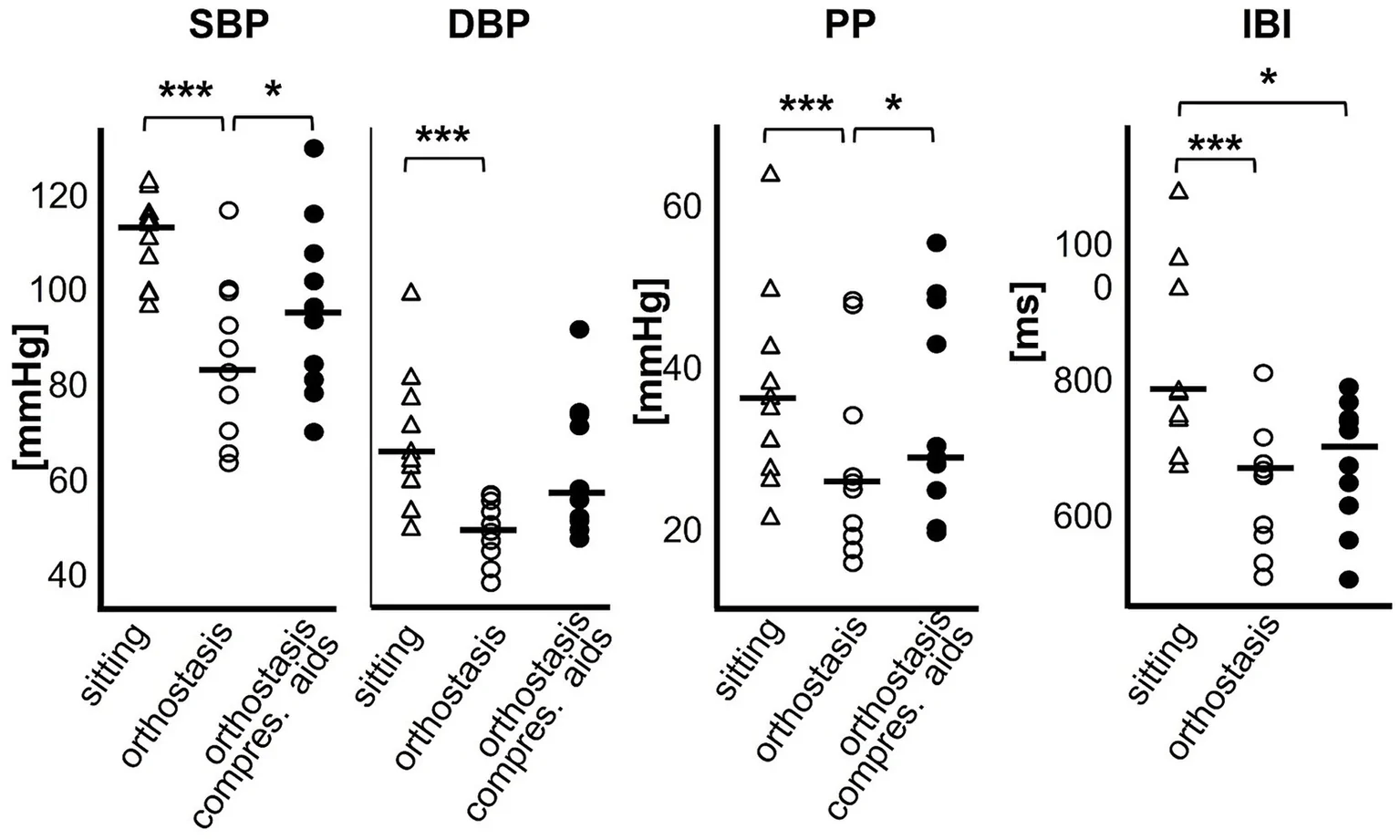
Mean values of systolic blood pressure (SBP), diastolic blood pressure (DBP), pulse pressure (PP), and inter-beat intervals (IBI) during sitting, during orthostasis without CA and during orthostasis with CA. Short horizontal line represents median. *, **, *** indicate statistical difference between phases with p values < 0.05, < 0.01, or < 0.001, respectively.

Without CA, verticalization was accompanied by a significant decrease in mean SBP, DBP, and PP values. Application of CA prevented the decrease in arterial BP values, resulting in a non-significant difference between phases 3 and 1 (Figure 1). Mean IBI interval was significantly decreased during orthostasis regardless of the CA application (Figure 1, right panel).

**Figure 2:**
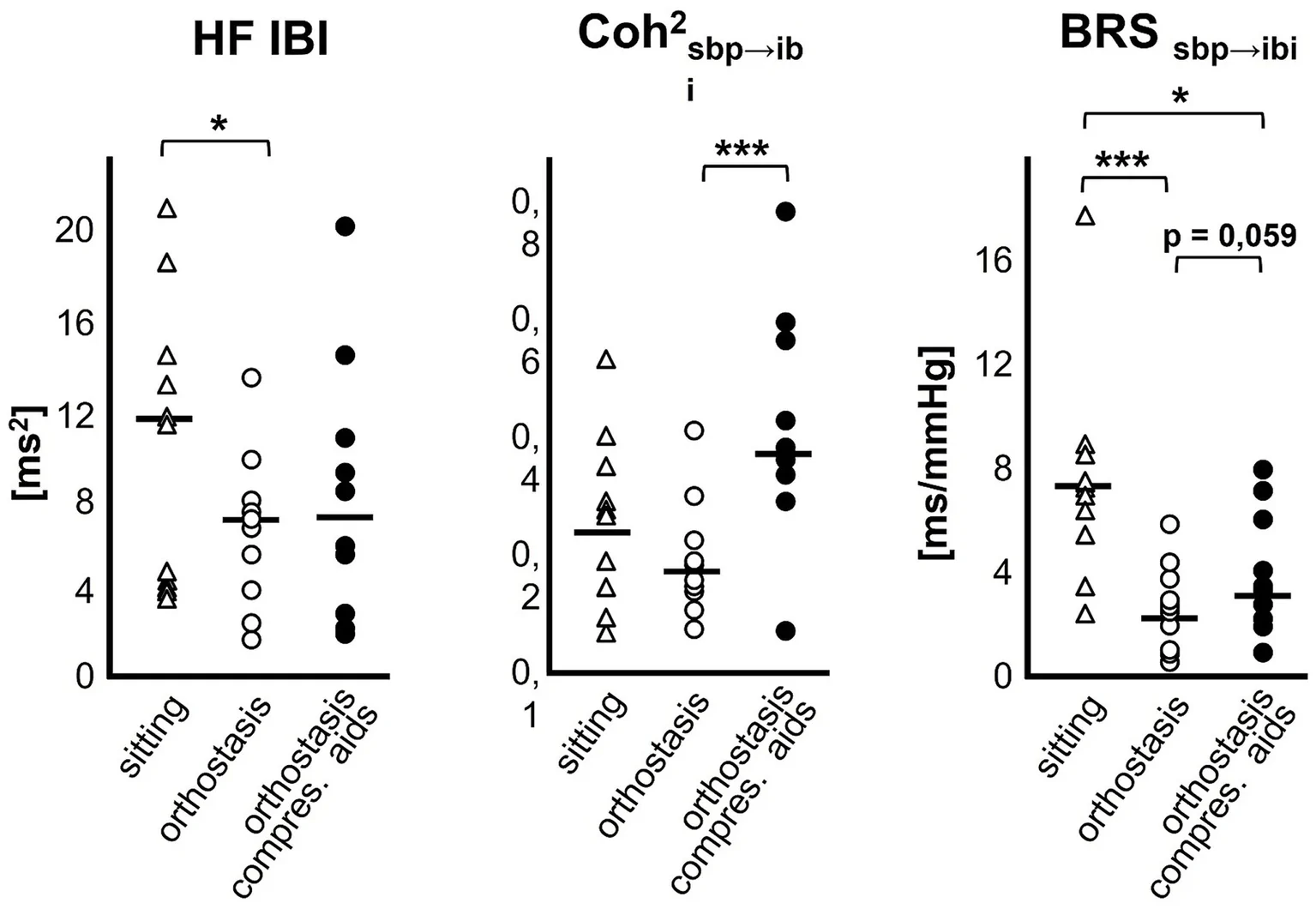
Spectral power in high frequency range 0.15–0.5 Hz (HF IBI), causal coherence (Coh2sbp → ibi) and baroreflex sensitivity (BRSsbp → ibi) during sitting, orthostasis without CA and orthostasis with applied CA. Short horizontal line represents median. *, **, *** indicate statistical difference between phases with *p* values < 0.05, < 0.01, or < 0.001, respectively.

Verticalization without CA was not associated with a significant change in Coh_sbp → ibi_. Using CA, coherence (Coh_sbp → ibi_) was increased during orthostasis (Figure 2, left panel). Compared to sitting, BRS_sbp→ibi_ was significantly lower during both verticalization phases with a tendency towards less pronounced decrease in this measure when CA were applied (verticalization without CA vs. with CA: *p* = 0.059) (Figure 2, right panel). Spectral power HF IBI was lower during orthostasis without compression aids in comparison to sitting values. This difference in HF IBI was not observed when compression aids were used (Figure 2).

## 4 Discussion

This study was focused on the effects of CA on basic cardiovascular parameters and parasympathetic autonomic control during orthostatic challenge in patients after cervical SCI. We hypothesized that an application of CA would not only prevent excessive BP decrease during orthostasis, but it would be also associated with less prominent autonomic nervous system response. Since the orthostasis shifts cardiovascular autonomic activity towards parasympathetic inhibition and sympathetic activation via baroreflex, lower BP decrease would be associated with less expressed parasympathetic inhibition, thus preserving functional reserve of cardiovascular autonomic control to further maneuvers/challenges (e.g., cognitive load, exercise) during orthostasis and decreasing the risk of OH.

There are two major findings of our study: prevention of BP decline during verticalization as a result of concomitant baroreflex mediated heart rate increase and CA application; abolished parasympathetic activity inhibition associated with orthostatic stress when using CA. Moreover, part of the subjects in our study reported improved tolerance to verticalization when the CA were used.

### 4.1 The effects of compression aids on BP response to orthostasis in cervical SCI patients

Arterial BP is determined by several cardiac and vascular factors, such as cardiac contraction strength significantly dependent on venous return, heart rate and peripheral vascular resistance. Several mechanisms influencing these variables are altered by cervical SCI (Claydon et al., 2006; Krassioukov and Claydon, 2006). Firstly, a loss of sympathetic supraspinal control over the major portion of vascular system leads to the excessive blood pooling in the vessels in the lower parts of the body (Weaver et al., 2012; Popa et al., 2010). Secondly, a loss of motor function in legs results in an impaired mechanism of skeletal muscle pump, thus compromising venous return (Chao and Cheing, 2008). Thirdly, lost motor control over the abdominal muscles and partially over the respiratory muscles in cervical SCI impairs the mechanism of venous return facilitation by increased pressure gradient between the chest and abdominal cavities during inspiratory phase (Huang et al., 1983). Taken together, above mentioned mechanisms contribute to the observed marked decrease in BP values (both SBP and DBP) during verticalization in cervical SCI patients. In addition, decreased PP often reflects a decreased stroke volume, further confirming these effects.

Compression aids help to increase venous return and thus could be beneficial in situations when venous return is markedly decreased, i.e., during orthostasis. While compression stockings prevent blood pooling in the lower extremities, abdominal binder supports mechanism of the respiratory pump, both contributing to improvement of venous return (West et al., 2012). Previous studies assessing the CA effects on hemodynamics in SCI patients demonstrated increased SBP at rest (Rimaud et al., 2007), reduced venous capacity (Rimaud et al., 2008), increased blood flow in the arm, and improved exercise performance (Rodrigues et al., 2022; Vaile et al., 2016). Although the beneficial effect of CA to attenuate BP drops during orthostasis was demonstrated in healthy subjects (Tezuka et al., 1997), the information about the effect of CA on BP response to verticalization in SCI patients is so far missing. Several authors have explored an effect of CA in SCI patients during exercise or body-position change during physiotherapy in order to improve performance or to limit post-exercise hypotension and/or OH (Popa et al., 2010; Hopman et al., 1992; Hopman et al., 1993; Kerk et al., 1995).

In our study, CA prevented BP drop during orthostasis, since no significant differences between sitting and orthostasis in SBP and DBP values were observed when CA were applied. Together with the preserved PP (reflecting non-significant change in stroke volume during verticalization) it indicates that compression aids increased venous return and cardiac filling, thus substituting missing vasoconstriction in the arterioles and veins in the legs.

### 4.2 The effects of compression aids on compensatory mechanisms to orthostasis in cervical SCI patients

Baroreflex is the most important short-term control mechanism preserving BP during orthostatic challenge. Baroreflex mediated response affects several important cardiovascular control targets, including heart rate, cardiac contractility, vascular resistance, and venous capacity (Krohova et al., 2020; Cooke et al., 1999; Fu et al., 2006). In cervical SCI patients, the cardiac chronotropic response—i.e., heart rate increase as a response to BP decrease—is mostly preserved, being to a major extent mediated by intact parasympathetic vagal nerves (Phillips et al., 2012). The absence of sympathetic cardiac chronotropic control in cervical SCI could potentially blunt baroreflex mediated heart rate increase. However, our results indicate that observed heart rate response prevented—together with an improvement of venous return by application of CA—the BP decrease during verticalization.

During orthostasis, the decrease of arterial BP sensed by baroreceptors results in cardiovascular parasympathetic activity inhibition and sympathetic activity increase. Indeed, the decreased magnitude of spectral power in HF band of heart rate variability in our study illustrates that parasympathetic control of cardiac pacemaker was decreased during verticalization and this decrease persisted with the application of CA. It corresponds to the persistence of IBI decrease (i.e., heart rate increase) with CA pinpointing the major mechanism of heart rate increase during orthostasis.

The behavior of baroreflex function related indices during our study protocol was more complex. Previous studies demonstrated that in addition to decreased BRS—an index of heart rate response magnitude to a unit change in BP—orthostasis is associated with an increased coherence between systolic BP and IBI in healthy human subjects (Krohova et al., 2020; Faes et al., 2013a; Faes et al., 2013b; Ondrusova et al., 2017; Porta et al., 2002; Porta et al., 2011). In agreement with previous studies on SCI patients, we found that BRS significantly decreased during verticalization. This decrease in BRS was suppressed by CA, i.e., there was a lower inhibition of reflex parasympathetic control when CA were used as compared to situation without them. Importantly, the BRS during verticalization with CA was still decreased as compared to sitting. This is in agreement with the decreased BRS associated with orthostasis in healthy subjects.

**Figure 3:**
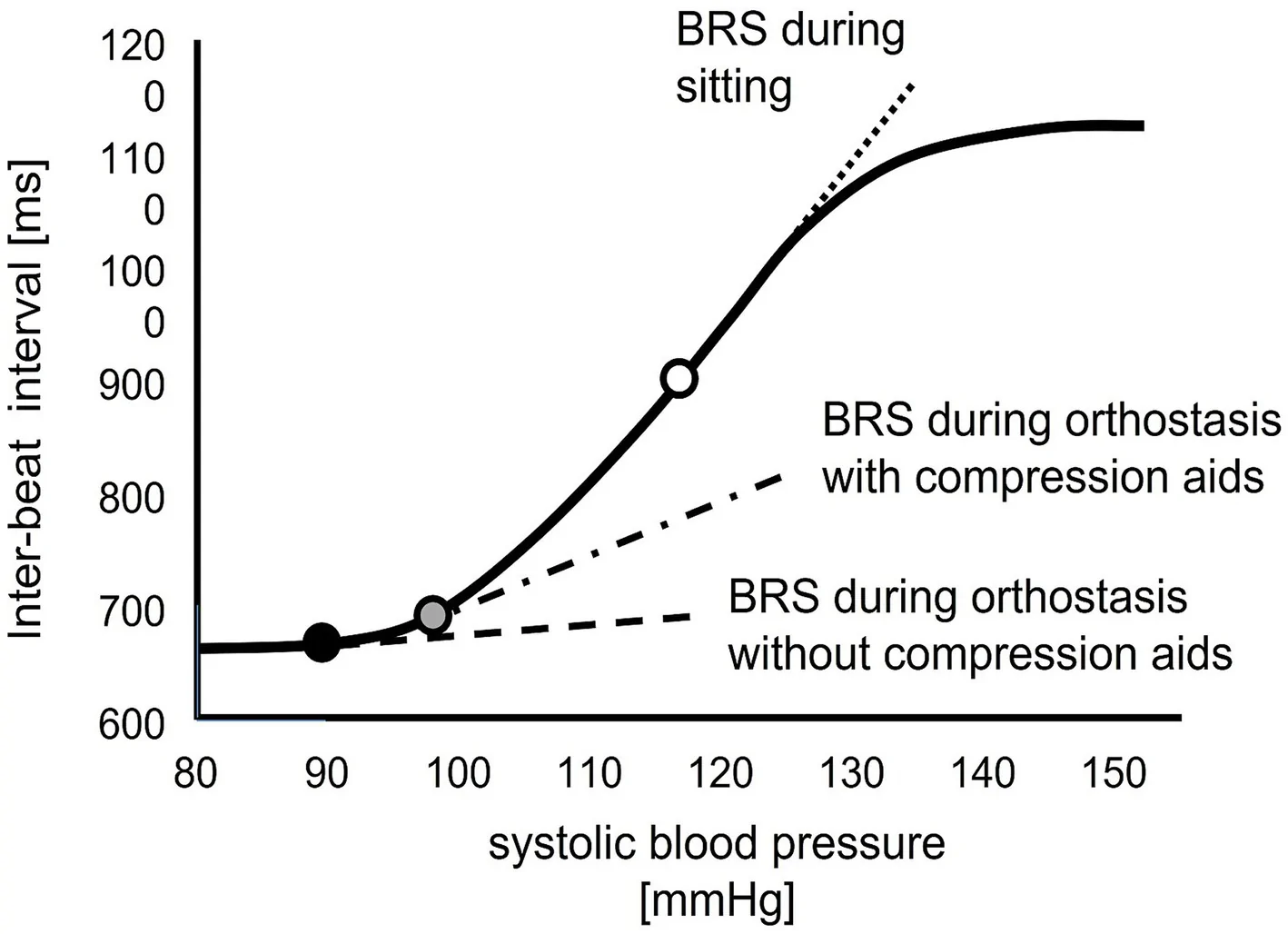
Dependence of IBI on SBP. BRS is defined as a slope of this curve. Three phases of measurement of cervical SCI patients are marked as assumed points on a curve—with white, gray and black points corresponding to sitting, verticalization with and verticalization without compression aids, respectively. Graph modified from (Korner et al., 1974; Parlow et al., 1995).

In cervical SCI patients in our study the expected increase in coherence values occurred only when CA were used. The missing increase in Coh2_sbp → ibi_ values during verticalization without CA indicates an impaired baroreflex function in these patients. One of possible explanations of the missing increase in coherence between SBP and IBI values and the marked decrease in BRS (accompanied with significant drop in SBP in cervical SCI patients during verticalization without CA) could be the shift of the operating point on the sigmoid baroreflex curve (Figure 3). The leftward shift on the baroreflex curve potentially leads to both lower BRS and coherence values. Alternatively, decreased BRS reflects an inhibition of parasympathetic activity during verticalization, further illustrated by the persistence of BRS decrease with the restoration of SBP values when CA are used. In summary, the response of baroreflex related measures (Coh2_sbp → ibi_ and BRS) to orthostatic challenge in cervical SCI patients using CA (but not in those without CA) was very similar to changes observed in previous studies on healthy subjects.

The studies on the effect of CA on cardiovascular control in SCI patients are scarce. To our best knowledge, there is only one study focused on this topic (Horiuchi et al., 2018) reporting that compression stockings increased HF power of heart rate variability during prolonged sitting (3 h) in patients with SCI as compared to those without CA. Our results extend this observation to verticalization of cervical SCI patients—we found no significant difference between HF power in heart rate variability between patients after verticalization wearing CA compared to sitting position. However, without CA, HF power significantly decreased during orthostasis, thus indicating a decreased parasympathetic activity.

Up to now, the effect of CA on baroreflex function has not been studied. Although it was found that compression strategies prevent against OH (Rodrigues et al., 2022; Krassioukov et al., 2009), physiological studies focused more specifically on the effect of CA on baroreflex function in SCI patients are missing. Our results demonstrate the improved baroreflex function in verticalized cervical SCI patients wearing CA. It indicates that the orthostatic challenge with CA requires lower parasympathetic inhibition and the cardiac chronotropic arm of the baroreflex is still able to respond to potential BP perturbations.

### 4.3 Subjective sensations of patients

Verticalization is an important part of rehabilitation after SCI, since it positively affects physical as well as mental health. In clinical and experimental testing, head-up tilt with milder slope is usually used. In physiotherapy of SCI patients, verticalizers such as Balance Thera-trainer are commonly used. Patients who experienced OH-related symptoms during verticalization (five patients, 50% of study sample), reported their significant improvement when wearing CA. Horiuchi et al. reported that compression stockings decreased negative sensation during 3 h lasting prolonged sitting in healthy subjects, which supports our observations in cervical SCI patients (Horiuchi et al., 2018).

The observed differences in orthostatic tolerance could be explained by the variations in the spinal cord completeness and the level of injury. Furthermore, interindividual variability in autonomic nervous system responses to orthostasis should be taken in consideration. The variations in the effectiveness of cerebral blood flow control mechanisms could also influence the presence of OH symptoms (Weaver et al., 2012). Several authors suggested that autoregulation of cerebral blood flow rather than systemic BP control could play a dominant role in the adaptation to OH in patients with SCI (Claydon et al., 2006; Bisharat et al., 2002; Gonzalez et al., 1991).

### 4.4 Physiological aspects

The results of our study point towards the importance of vascular baroreflex response mediated by sympathetic nervous system during orthostatic challenge. It includes both vasoconstriction in the resistance vessels (mostly arterioles) and in the capacitance vessels in the splanchnic, musculo-cutaneous, and renal vascular beds. Without these compensatory mechanisms or their surrogate (e.g., effects of CA), the BP and baroreflex function markedly decreased. Having in mind the well preserved cardiac chronotropic baroreflex response—an increase in heart rate during verticalization, it shows that heart rate response itself is not able to fully compensate for BP decrease during orthostasis (Smit et al., 1999). Interestingly, the CA substituted sympathetic control of capacitance vessels (venules and veins) resulting in complete compensation of BP drop associated with verticalization.

From the pathophysiological point of view, decreased BRS together with decreased cardiac chronotropic baroreflex coherence reflect a potentially dangerous baroreflex control system state, ineffectively dampening further BP perturbations and potentially leading to orthostatic syncope. The baroreflex control during verticalization with CA in cervical SCI patients is more physiological and less “on the limit” (Figure 3). Baroreflex analysis during verticalization with CA indicates that cardiovascular reflex control enables to compensate better for any BP change as compared to cervical SCI patients without CA. It could be one of the mechanisms of better subjective tolerance of verticalization in cervical SCI patients wearing CA.

## 5 Study limitations

Study limitations should be acknowledged. First, the study included only ten participants. Recruiting a larger cohort of patients with traumatic spinal cord injury localized exclusively to the C4-C7 segment would either require a substantially longer recruitment period or multicenter collaboration. Moreover, the relatively small number of such injuries fortunately reflects their low incidence in the population. The limited sample size is also related to the second limitation, namely the considerable variability in the time elapsed since injury. Because all participants were recruited from a single specialized center and time since injury was not used as an exclusion criterion, all patients meeting the remaining eligibility criteria were included.

Third, the cohort showed a marked gender imbalance, consisting of nine men and one woman. This distribution reflects the well-documented predominance of traumatic spinal cord injuries among males, which is commonly attributed to a higher frequency of risk-taking behaviors and exposure to high-risk activities. Fourth, the study was restricted to lesions located between C4 and C7. This region was selected because the majority of traumatic spinal cord injuries occur in the lower cervical spine, making it possible to recruit a sufficiently homogeneous patient cohort. Finally, the assessment of lesion level and severity was based on somatosensory deficit evaluation. Although clinically relevant, this approach may not always precisely correspond to the anatomical extent of spinal cord damage and therefore represents an important methodological limitation of the study.

## 6 Conclusion

Our results indicate that CA markedly improve cardiovascular response to verticalization by minimizing the BP drop by partial substitution of a missing sympathetic vascular response to orthostasis (vasoconstriction in resistance and capacitance vessels). Parasympathetic nervous system control in cervical SCI is less suppressed, which results in the preserved baroreflex function as another potentially important mechanism of OH prevention. Patients suffering from OH symptoms reported significant improvement of their orthostatic tolerance when wearing CA. Since orthostatic intolerance could significantly impair physiotherapy and limit the patient’s daily activities, our results evidence that CA are beneficial as a non-pharmacological prevention of OH in cervical SCI patients. The use of CA is thus extending the possibilities of the rehabilitation and improving the quality of life in cervical SCI patients.

## Data Availability

The raw data supporting the conclusions of this article will be made available by the authors, without undue reservation.
